# Herbal Medicine (HM) among pharmacy professionals working in drug retail outlets in Asmara, Eritrea: knowledge, attitude and prevalence of use

**DOI:** 10.1186/s12906-022-03698-8

**Published:** 2022-08-12

**Authors:** Alemseghed Goitom, Feruz Yemane, Mussie Tsegay, Amanuel Kifleyesus, Senai Mihreteab Siele, Eyasu H. Tesfamariam, Nuru Abdu

**Affiliations:** 1 Department of Medical Sciences, Pharmacy Unit, Orotta College of Medicine and Health Sciences, Asmara, Eritrea; 2 Biostatistics and Epidemiology, Department of Statistics, College of Sciences, Mai-Nefhi, Eritrea; 3 Department of Pharmacy, Adi-Tekelezan Community Hospital, Adi-Tekelezan, Eritrea

**Keywords:** Knowledge, Attitude, Prevalence of use, Herbal medicine, Pharmacy professionals, Drug retail outlets, Eritrea

## Abstract

**Background:**

Globally, the usage of herbal medicines (HMs) is increasingly growing in treating and preventing various ailments. Although, HMs play a vital role in healthcare, concerns have been raised over their safety. Since pharmacy professionals are at the right position to provide patients with evidence-based information on herbals, they should be knowledgeable enough on the subject matter. Thus, the aim of the study was to assess the knowledge, attitude and prevalence of HMs use and its associated factors among pharmacy professionals.

**Method:**

An analytical cross-sectional study was conducted among pharmacy professionals working in drug retail outlets in Asmara (the capital city of Eritrea). A census design was employed and data were collected through face-to-face interview. Data were entered and analyzed using Census and Survey Processing System (version-7.2) and IBM Statistical Package for Social Sciences (version 26), respectively. Descriptive and analytical statistics including Mann-Whitney U test/Kruskal-Wallis test and logistic regression were employed. *P*-values less than 0.05 were considered as significant.

**Results:**

A total of 50 pharmacy professionals (90.9% response rate) were enrolled in the study. Majority of them (62%) were males and two-thirds had a bachelor’s degree. The overall median (Interquartile range, IQR) knowledge score was found to be 24 (12.16) out of 100 with a minimum score of 8 and maximum score of 53. Generally, the median (IQR) attitude score was 70.4 (4.2) out of 100. Majority (78%) of them had used HM for self-treatment. Only religion was found to be a significant determinant of knowledge on indication of HMs (*p* = 0.015), while attitude score was independent of the potential determining factors. Moreover, prevalence of use was significantly associated with pharmacy ownership (Adjusted Odds Ratio (AOR) =14.44, 95%Confidence Interval (CI): 1.67, 124.52) as well as with the percentage attitude score (AOR = 0.632, 95%CI: 0.41, 0.96) at multivariable level.

**Conclusion:**

Generally, the overall knowledge score of pharmacy professionals was low. However, they possessed positive attitude towards herbal medicines. Besides, there was prevalent usage of herbal medicine for self-treatment. This outcome triggers the need for educational courses and workshops centered on herbal medicine.

**Supplementary Information:**

The online version contains supplementary material available at 10.1186/s12906-022-03698-8.

## Introduction

Herbal medicines are commonly used worldwide in treating and preventing various ailments and play a vital role in the healthcare system. According to World Health Organization (WHO), herbal medicine is defined as a set that includes herbs, herbal materials, herbal preparations and finished herbal products that contain, as active ingredients, parts of plants, other plant materials or combinations thereof. In certain countries, herbal medicines may usually contain natural organic or inorganic active ingredients that are not of plant origin [1].

Globally, the use of herbal medicines is increasingly growing in parallel to the greater demand by consumers. WHO estimates that 80% of the developing world’s population meets some aspects of their primary healthcare need through herbal medicines. This is owed to their high acceptance by patients who appreciate their active role and perceived autonomy in choosing herbal medicines and they also do perceive them to be highly efficacious and safe [2]. As a result, patients are highly encouraged to self-medicate with HMs.

This rise in prevalence of use, however, has raised an increasing safety concern of these preparations because not only are they inadequately studied but there is also lack of suitable quality controls, inadequate labeling and absence of appropriate patient information of herbal medications [3]. In addition, a number of adverse events, drug interactions and death are on rise which further intensifies their safety concerns. In recent years, the increasing use and the aforementioned safety issues have triggered healthcare professionals worldwide to take interest in HMs. Pharmacy professionals, in particular, if knowledgeable enough, are on the right position to provide appropriate evidence-based information on the safe use of these preparations. As per the WHO traditional and complementary global report, a total of 199 member states had reported the sale of herbal medicines in drug retail outlets as of 2012 [1]. This trend is expected to increase in the coming years making pharmacy professionals occupy the center stage in the sale and safe use of HMs.

In Eritrea, traditional medicine has been an important part of the culture and tradition of the people for centuries, and despite the accessibility of conventional medicine to the majority of the population, Eritreans still rely on traditional medicine practice and products [4]. HM use is common as well, though it is not appropriately documented. The rationale behind wide spread use of HM is due to perceived efficacy, safety and cultural acceptability [5]. For this reason, the National Medicines and Food Administration (NMFA), since the establishment of the Traditional Medicine Unit (TMU) in 2012, has been endeavoring in regulation of TM and ensuring the safety, quality and efficacy of TM practices and products [1].

Despite, the fact that herbal medicine use is on the rise, there is no systematic regulation on the practice. In Eritrea, there is no legal basis for the regulation of TM in general and herbal products specifically. Nevertheless, the reality is much more different in that contraband herbal products exist in the market bypassing the regulatory requirements. This practice predisposes consumers to hazards.

Be that as it may, there is lack of information on whether pharmacy professionals have a sound knowledge on the subject matter to safeguard or counsel consumers. In Eritrea, pharmacy professionals do take undergraduate courses related to HM. However, currently there are no researches in Eritrea to confirm whether the undergraduate courses influence the knowledge of the pharmacy professionals on herbal medicine. Moreover, as consumers revert to drug retail outlets for any information on any type of medication, there is no information to determine whether pharmacy professionals are knowledgeable enough on HM to provide proper counseling and make recommendations, despite taking undergraduate courses related to HM. Hence, this study allowed the researchers to partially determine whether these courses were helpful to the professionals in affecting their current knowledge. The aim of the study was to assess the knowledge, attitude and prevalence of use of HMs and its associated factors among pharmacy professionals.

## Methods

### Study design and setting

An analytical cross-sectional study with a quantitative approach was conducted in all drug retail outlets in Asmara (the capital city of Eritrea) between April and June, 2021. In Eritrea, there are a total of six administrative regions (Maekel, Anseba, Gash-Barka, Debub, Debubawi Keih Bahri and Semenawi Keih Bahri). A total of 84 drug retail outlets are available across the country. In Asmara, which is located within the Maekel region, there were 44 drug retail outlets (12 drug shops and 32 community pharmacies) during the study period. Six of the community pharmacies are governmental, also known as chain pharmacies. Two of the chain pharmacies are located inside Orotta and Halibet National Referral Hospitals.

### Source and study population

All pharmacy professionals who worked in all the drug retail outlets in Asmara were the source population for this study. Thus, the complete enumeration of the pharmacy professionals who were involved in drug retail outlets was conducted and those who had expressed willingness to be included in the study formed the study participants. Pharmacy professionals who worked in other areas apart from drug retail outlets and interns were excluded from the study.

### Data collection tool

A self-designed, structured and interview-based questionnaire (Additional file 1) that was prepared upon a review of similar published studies [2, 6, 7] was used to collect data. The questionnaire had the following four sections: questions that capture socio-demographic and background characteristics, knowledge, attitude and prevalence of use of the respondents’. Knowledge was measured using 74 items with four sub-sections designed to assess the general awareness of pharmacy professionals on herbal medicines’ (HM) indications, interactions, contraindications and precautions and adverse effects/side-effects. In order to avoid confusion by the respondents’, the names of the selected herbs were written in both their scientific (taxonomical name) and local name (in Tigrigna language). Attitude was measured using a 25-items related to the beneficial effects and safety of herbal medicines, their perceived role in dispensing herbal medicines, the use of herbal medicines by the public and the continuous educational programs centered on herbal medicines. Prevalence of use was measured using 9-items related to the personal use of herbal medicines by the respondents’.

### Data collection procedure

The data collectors explained the purpose of the study to the subjects and those who gave consent were recruited in the study. Then, questionnaires were filled through a face-to-face interview. Study subjects who were not able to participate at the time of the collection of data, due to absence at the work place, were asked if they could participate in another time and those who seemed highly doubtful or not willing to take part in the study, were considered as non-respondents.

### Variables

The knowledge, attitude and prevalence of use of herbal medicines among the pharmacy professionals were considered as dependent variables and the socio-demographic and other background characteristics such as age, gender, religion, marital status, educational level, pharmacy ownership, ever taken a training, years of working experience and type of drug retail outlet were considered as independent variables for this study.

### Variable measurement

Knowledge section consisted of 74 items. The sub-sections of knowledge such as indication, interaction, contraindication/precautions and adverse event consisted of 47, 7, 5 and 15 items, respectively. A score of 1 was given to the correct answers and 0 to the wrong ones. Then the assigned scores were added and divided by the total number of items and further multiplied by 100 in order to get the composite knowledge score for the overall knowledge and the specific sub-sections of knowledge. The attitude consisted of 25 items. It was measured using 5-point likert scale as strongly agree, agree, neutral, disagree and strongly disagree. For the purpose of descriptive analysis, merging was done for ‘strongly agree’ and ‘agree’ as one group; and ‘disagree’ and ‘strongly disagree’ as another; leaving ‘neutral’ as third separate group. The total score was divided by 125 (a maximum of 5 points for each statement) and then multiplied by 100 in order to get the overall attitude percentage score. Response for a negatively worded item was at first reverted to get a composite attitude score.

### Data quality control

To ensure face and content validity, the questionnaire was subjected for peer-review of selected experts in the fields of pharmacy and epidemiology, including experts from the National Traditional Medicine Unit of the Ministry of Health. Additionally, a botanist was approached in the selection of herbs. The content validity index (CVI) was computed at item level, and all items rated as relevant were included to make the I-CVI of the final questionnaire one. The questionnaire was then changed as per the provided comments and further subjected to pre-test. The pre-test was conducted on 6 participants from 17 to 19 March, 2021 in drug retail outlets outside the study sites. It was aimed at checking the questionnaire’s appropriateness, completeness, flow of questions, skip patterns as well as habituation of data collectors, approximation of the time for completion of the questionnaire and accuracy of the questions. Prior to the pre-test, a two-day orientation workshop was given to the data collectors. As per the experiences earned during the pre-test, the questionnaire was revised and used for the actual data collection.

### Statistical analysis

The collected data was double entered on CSPro (version 7.2) to minimize the keying errors. The entered data was exported to SPSS (version 26) for statistical analysis. Descriptive summaries of the percentage scores of knowledge, and attitude were done using median (IQR) after assessing the normality of the quantitative variables. The potential difference in percentage scores of knowledge and attitude across the categories of demographic variables was assessed using Mann-Whitney U test (for variables with two categories) and Kruskal-Wallis test (for variables with more than two categories). Fisher’s exact test and bivariate logistic regression was used to assess the association between the demographic variables and knowledge as well as attitude scores with the prevalence of usage in herbal medicine among the pharmacy professionals. Variables that were significant at bivariate logistic regression and Fisher’s exact test were retained for multiple logistic regressions. Crude odds ratios (COR) and adjusted odds ratio (AOR) with their 95% confidence intervals (CIs) were reported for the multiple logistic regressions. A *p*-value of 0.05 or less was used as cut off level for statistical significance.

### Operational definitions

**Complementary and Alternative medicine (CAM):** the two terms used interchangeably with traditional medicines, often refer to traditional medicine that has been adopted by other populations (outside its indigenous culture). They are sometimes used to refer to health care that is considered supplementary to allopathic (conventional) medicine [1].

**Conventional Medicine:** refers to the broad category of medical practice that is sometimes called allopathic medicine, western medicine, biomedicine, scientific medicine or modern medicine [1].

**Herbal medicine (HM):** includes herbs, herbal materials, herbal preparations and finished herbal products which contains as active ingredients parts of plants or other plant materials or combinations [1].

**Traditional medicine (TM):** is the sum total of knowledge, skills and practices based on the theories, beliefs and experiences indigenous to different cultures, whether explicable or not used in the maintenance of health and the prevention, diagnosis, improvement or treatment of physical and mental illness [1].

**Drug retail outlet:** a retail outlet where medicines are sold under the supervision of a pharmacy technician in the case of drug shops or under the supervision of a pharmacist in the case of a community pharmacy.

## Results

### Socio-demographic and background characteristics

A total of 55 pharmacy professionals were approached by the data collectors, where 50 of them gave consent to participate in the study with a response rate of 90.9%. The median (IQR) age of the respondents was 39.5 (20) with a minimum age of 21 and maximum of 77. Most of the respondents (62%) were males and with a bachelor’s degree in pharmacy (64%). About 12% of the respondents said that they had either previously received training or participated at a workshop on herbal medicines. The years of experience of the professionals’ in drug retail outlets and in the field of pharmacy ranged from 1 to 48 years. Majority of the respondents (68%) worked in private retail outlets. Moreover, 84% of the total respondents worked in community pharmacies (Table 1).

**Table 1:** Socio-demographic and background characteristics of the study population (*N* = 50)

Variable	Category	Number	Percent
Age (Md, IQR: 39.5; 20)	20-29	10	20
	30-39	15	30
	40-49	12	24
	50 and above	13	26
Sex	Male	31	62
	Female	19	38
Pharmacy ownership	Owner	9	18
	Employee	41	82
Educational level	Diploma	15	30
	BPharm	32	64
	MSc	3	6
	PhD	0	0
Marital status	Single	13	26
	Married	34	68
	Separated	3	6
	Widowed	0	0
Training on herbal medicine	Yes	6	12
	No	44	88
Religion	Christian	46	92
	Muslim	4	8
Years of experience in drug retail outlets	1-9	22	44
	10-19	16	32
	20-29	9	18
	30-39	1	2
	40 and above	2	4
Years of experience in the pharmacy field	1-9	18	36
	10-19	18	36
	20-29	3	6
	30-39	8	16
	40 or above	3	6
Type of drug retail outlet by privacy	Yes	16	32
	No	34	68
Type of drug retail outlet	Drug shop	8	16
	Pharmacy	42	84

*Md* Median, *IQR* Interquartile range

### Knowledge of herbal medicines and its determinants among pharmacy professionals

#### Herbal medicine knowledge on indication

Most (64%) of the respondents correctly indicated the use of *Chaenopodium album* in hypertension and nearly half (52%) of the respondents correctly indicated the uses of *Citrus lemon* in digestive problems, *Azadirachta indica* use as an insecticide (54%), *Carica papaya* in constipation (52%) (Table 2). The median (IQR) knowledge score for indications of herbal medicines was 27.65 (14.89) out of 100. Mann-Whitney U test showed that Christians (Md = 27.66, IQR = 15.43) had significantly higher (*p* = 0.015) percentage knowledge score on the indication of herbal medicines as compared to Muslims (Md = 15.96, IQR = 8.51). However, a statistically insignificant knowledge differences of indication were also observed across the categories of drug retail outlet type by privacy (*p* = 0.08), drug retail outlet type (*p* = 0.568), gender (*p* = 0.288), educational level (*p* = 0.909), marital status (*p* = 0.873), pharmacy ownership (*p* = 0.196), ever taken training or workshop on HMs (*p* = 0.519), age (*p* = 0.287), work experience in drug retail outlet (*p* = 0.284) and overall work experience in the field of pharmacy (*p* = 0.066) (Additional file 2).

**Table 2:** Pharmacy professionals’ knowledge on indication of herbal medicine in Asmara, Eritrea, 2021 (*N* = 50)

Herbal medicine	Indications	Correct answers	
		Number	Percent
*Aloe camperi*	Fungal infection	15	30
	Impotency	0	0
	Abdominal pain	5	10
*Zingiber officinale*	Emesis	13	26
	Flatulence	10	20
	Migraine	8	16
	Common cold and flu	25	20
*Meriandra dianthera*	Hypertension	14	28
	Diabetes	4	8
*Schinus molle*	Diarrhea	3	6
	Common cold	10	20
	Cough	9	18
	Abdominal pain	14	28
*Ruta chalepensis*	Common cold	25	50
	Cough	21	42
	Abdominal pain	19	38
*Senna singueana*	Vomiting	3	6
	Loss of appetite	0	0
	Hepatitis	1	2
*Azadirachta indica*	Hemorrhoids	0	0
	Fungal infections	7	14
	Insects	27	54
*Allium sativum*	Hypertension	20	40
	Malaria	2	4
	Asthma	13	26
	Thrombosis	7	14
	Common cold and flu	3	6
*Carica papaya*	Diabetes	2	4
	Amoeba infection	5	10
	Thyroid fever	0	0
	Malaria	1	2
	Constipation	26	52
*Citrus lemon*	Gastritis	3	6
	Digestive problems	26	52
*Aloe elegance*	Diabetes	12	24
	Bacterial infection	17	34
*Chaenopodium album*	Hypertension	32	64
	Burns	0	0
	Wounds	1	2
*Trigonella foenum-graecum*	Hypertension	6	12
	Diabetes	8	16
	Asthma	3	6
	Abdominal pain	19	38

#### Herbal medicine knowledge on interactions

About 38% of the respondents’ knew that *Allium sativum* interacts with ACE-inhibitors causing marked hypotension and it also interacts beneficially with fish oils to lower blood lipids. Moreover, three-fifth of the respondents’ (30%) knew that *Zingiber officinale* interacts with pharmacologic effects of warfarin and that it can decrease the metabolism of caffeine (22%). Fourteen percent knew that *Schinus molle* might interact with theophylline. However, only 6% of the respondents knew that the concomitant use of *Senna singueana* and conventional corticosteroids might increase the risk of hypokalemia. Likewise, only four out of the total fifty respondents knew that *Aloe camperi* juice reduces blood-glucose levels in patients with diabetes taking hypoglycemic agents such as glibenclamide.

The median (IQR) knowledge score for interactions of herbal medicines was 21.42 (42.86) out of 100. The median knowledge score of the associations of the interactions of herbal medicines across the categories of drug retail outlet by privacy (*p* = 0.20), drug retail outlet type (*p* = 0.321), gender (*p* = 0.758), religion (*p* = 0.70), educational level (*p* = 0.929), marital status (*p* = 0.477), pharmacy ownership (*p* = 0.331), ever taken training or workshop (*p* = 0.806), age (*p* = 0.174), work experience in drug retail outlet (*p* = 0.333) and overall work experience in pharmacy field (*p* = 0.138) revealed no significant differences (Additional file 3).

#### Herbal medicine knowledge contraindications and precautions

Sixty-two percent of the pharmacy professionals’ correctly answered that *Azadirachta indica* seeds are contraindicated in children. About 40% of them knew that *Allium sativum* is contra-indicated in people with allergies. Moreover, 34 % of them knew that *Zingiber officinale* is contraindicated in individuals with gallstones. However, only 22% of the respondents’ correctly answered that *Aloe camperi* should be used with precaution in patients with diabetes mellitus and that *Trigonella foenum-graecum* is contra-indicated in pregnancy.

The median (IQR) knowledge score for contraindications and precautions of herbal medicines was 40 (40) out of 100. Associations of the median knowledge scores of the contraindications and precautions across the categories of drug retail outlet by privacy (*p* = 0.794), drug retail outlet type (*p* = 0.472), gender (*p* = 0.933), religion (*p* = 0.526), educational level (*p* = 0.322), marital status (*p* = 0.628), pharmacy ownership (*p* = 0.937), ever taken training or workshop (*p* = 0.445), age (*p* = 0.553), work experience in drug retail outlet (*p* = 0.901) and overall work experience in pharmacy field (*p* = 0.926) revealed no significant differences (Additional file 4).

#### Herbal medicine knowledge on adverse events and/or side-effects

Sixty-percent of the respondents correctly stated that *Zingiber officinale* causes heartburn. About 46% of the respondents correctly indicated that *Allium sativum* causes indigestion. On the other hand, none of the respondents knew that long term use of *Azadirachta indica* causes miscarriage and infertility (Table 3).

**Table 3:** Pharmacy professionals’ knowledge on adverse events and/or side-effects of herbal medicine in Asmara, Eritrea, 2021 (*N* = 50)

Statements	Adverse events and/or side-effects	Correct answers	
		Number	Percent
*Allium sativum* may cause	Indigestion	23	46
	Hypersensitivity reactions (contact dermatitis and asthma)	11	22
*Aloe camperi* juice can cause	Abdominal cramps	8	16
	Diarrhea	9	18
*Zingiber officinale* causes	Heartburn	30	60
	Allergic reactions (rashes)	5	10
*Trigonella foenum-graecum* causes	Abdominal distension	5	10
	Diarrhea	6	12
	Dyspepsia	4	8
	Flatulence	13	26
Long term use of *Azadirachta indica* may cause	Kidney injury	2	4
	Liver injury	6	12
	Miscarriages	0	0
	Infertility	0	0
	Hypoglycemia	3	6

The median (IQR) knowledge score for adverse events of herbal medicines was 13.33 (20.00) out of 100. There was no statistically significant association of the median knowledge score for adverse events across the categories of different socio-demographic and other background variables (Additional file 5). Generally, the median of overall percentage knowledge score (IQR) was 24.00 (12.16) out of 100.

### Attitude of pharmacy professionals on herbal medicines and its associated factors

Almost all of the respondents (96%) believed that herbal medicines have beneficial effects. Moreover, majority of them (82%) believed that herbal medicines have a high acceptance by the public. While, 62% agreed that herbals have significant interaction with conventional medicine, 58% of them disagreed that herbal medicines have fewer side effects than conventional medicines. More than half of them (58%) agreed that herbal medicines are readily contaminated and cannot be used safely. Nonetheless, 98% admitted that pharmacy professionals need to be educated on herbal medicine use and their side effect.

Majority (84%) had a positive attitude on the item “Pharmacy professionals are in unique position to provide evidence-based information regarding herbal medicines to help patients and customers make safe decisions about their use” and 76% of them agreed that they should be in the right position to advice patients on herbal medicines. Most of the respondents’ (72%) said that finished herbal products should be sold only in a drug retail outlet and that they must be incorporated with the conventional health care system to avoid self-medication. In addition, about half of the respondents (54%) rejected the statement that “carrying herbal medications may have a negative influence on their pharmacy’s image”. However, 96% of the respondents were complacent with the statement “educational courses on herbal medicines centered on patient care (counseling and dispensing) must be given to pharmacy professionals”. More than 90% agreed that indigenous knowledge from herbalists should be integrated in the guidelines of use and that workshops and training shall be provided to pharmacy professionals and herbalists to improve their inter-professional relationship (Table 4).

**Table 4:** Pharmacy professionals’ attitude towards herbal medicine, Asmara, Eritrea, 2021 (*N* = 50)

Statement	Agree/Strongly agree (%)	Neutral (%)	Disagree/Strongly disagree (%)
Herbal medicines have beneficial effects.	48 (96)	1 (2)	1 (2)
Herbal medicines are readily contaminated and cannot be used safely.	29 (58)	3 (6)	18 (39)
Herbal medicines are relatively safer, because they are natural.	13 (26)	5 (10)	32 (64)
Herbal medicines have fewer side effects than conventional medicines.	14 (28)	7 (14)	29 (58)
Herbal medicines have high qualities (in terms of active ingredients).	35 (70)	6 (12)	9 (18)
Herbal medicines are as efficacious as conventional medicines.	21 (42)	9 (18)	20 (40)
Herbal medicines have placebo effects.	32 (64)	11 (22)	7 (14)
Herbal medicines have relatively fewer interactions in comparison with conventional medicines.	18 (36)	11 (22)	21 (42)
Herbal medicines have relatively fewer contra-indications in comparison with conventional medicines.	16 (32)	14 (28)	20 (40)
Herbal medicines have significant interactions with conventional medicine.	31 (62)	7 (14)	12 (24)
Pharmacy professionals are in unique position to provide evidence-based information regarding herbal medicines to help patients and customers make safe decisions about their use.	42 (84)	3 (9)	5 (10)
Pharmacy professionals are the right persons to advise and educate people on herbal products use.	38 (76)	4 (8)	8 (16)
Herbal medications (finished herbal products) should be sold only in a pharmacy.	36 (72)	5 (10)	9 (18)
Carrying herbal medications may have a negative influence on a pharmacy’s image.	18 (36)	5 (10)	27 (54)
Herbals should be sold in pharmacies under a pharmacy professionals’ supervision.	37 (74)	6 (12)	7 (14)
Only registered pre-packaged herbal medicines should be available in community pharmacies.	42 (84)	2 (4)	6 (12)
Herbal medications are a threat to public health.	18 (36)	2 (4)	30 (60)
Herbal medicines have a high acceptance by the public.	41 (82)	3 (6)	6 (12)
Herbals have a positive impact on public health.	35 (70)	6 (12)	9 (18)
Herbal medicines should be incorporated along with the conventional medicine in the health care-service, in order to avoid self-medications by the public.	37 (74)	6 (12)	7 (14)
Educational courses on herbal medicines centered on patient care (counseling and dispensing) have to be provided to pharmacy professionals.	48 (96)	0	2 (4)
Indigenous knowledge from herbalists should be integrated for guidelines of use.	47 (94)	1 (2)	2 (4)
Pharmacy professionals need to be educated on herbal medicine use and their side effects.	49 (98)	0	1 (2)
The use of herbal medicines is an economic alternative to conventional medicines.	28 (56)	8 (16)	14 (28)
Workshops and trainings should be provided in order to establish inter-professional relationships between herbalists and pharmacy professionals.	49 (98)		1 (2)

The median (IQR) attitude percentage score was 70.4 (4.20) out of 100. A statistically insignificant attitude differences were detected across the categories of drug retail outlet type by privacy (*p* = 0.566), drug retail outlet type (*p* = 0.1), gender (*p* = 0.718), religion (*p =* 0.760), educational level (*p* = 0.565), marital status (*p* = 0.243), pharmacy ownership (*p* = 0.543), ever taken training or workshop (*p* = 0.182), age (*p* = 0.276), work experience in drug retail outlet (*p* = 0.204) and overall work experience in the field of pharmacy (*p* = 0.141) (Table 5).

**Table 5:** Determinants of pharmacy professionals’ attitude on herbal medicine across the categories of socio-demographic and other background characteristics at bivariate level, Asmara, Eritrea, 2021 (*N* = 50)

**Variable**	**category**	**Median (IQR)**	**Mann-Whitney Z/Kruskal-Wallis χ**^**2**^	***p*-value**
Type of drug retail outlets by privacy	Governmental	70 (5.4)	−0.57	0.566
	Private	70.4 (4.0)		
Type of drug retail outlets	Drug shop	68.8 (3.60)	−1.65	0.1
	Pharmacy	70.4 (4.20)		
Sex	Male	70.4 (4.0)	−0.36	0.718
	Female	70.4 (4.80)		
Religion	Christian	70.4 (4.80)	−0.31	0.760
	Muslim	70 (1.40)		
Educational level	Diploma	69.6 (4.0)	1.14	0.565
	BPharm	70.4 (4.0)		
	MSc	72 (−)^a^		
Marital status	Single	72.8 (6.0)	2.83	0.243
	Married	70.4 (4.0)		
	Separated	69.6 (−)^a^		
Pharmacy ownership	Owner	69.6 (4.0)	−0.61	0.543
	Employee	70.4 (4.4)		
Training or workshop on herbal medicines	Yes	68 (7.60)	−1.33	0.182
	No	70.4 (4.0)		
**Variables**			**r**_**s**_	***p*-value**
Age			−0.157	0.276
Work experience (in retail pharmacy outlet)			−0.183	0.204
Overall work experience (pharmacy field)			−0.211	0.141

*IQR* Interquartile range, *Z* Z score

^a^Too few data to get the IQR, χ^2^: Chi-square, r_s_: Spearman rank correlation

### Prevalence of use of herbal medicines by pharmacy professionals and its determinants

Almost three-quarters of the respondents (78%) used herbal medicines for self-treatment in their lifetime. Furthermore, 34% of them did not recommend customers to use herbal medicines for self-treatment. Only a fifth of the respondents (20%) seek scientific references before making recommendations or before personal use of herbal medicines (Table 6).

**Table 6:** Prevalence of herbal medicine use among pharmacy professionals in Asmara, Eritrea, 2021 (*N* = 50)

Questions	Responses	Frequency	Percent
Have you ever used herbal medicines for self-treatment?	Yes	39	78
	No	8	16
	I don’t remember	3	6
Have you ever used herbal medicines for minor ailments (common cold, scratches, tonsillitis...etc)?	Yes	41	82
	No	8	16
	I don’t remember	1	2
Have you ever used herbal medicines to relieve some diseases on the recommendation of other conventional health professionals, herbalists or layman?	Yes	20	40
	No	30	60
Do you have any chronic diseases?	Yes	10	20
	No	40	80
Do you use herbal medicines to manage your chronic diseases?	Often	4	8
	Sometimes	3	6
	Never	3	6
Do you use herbal medicines to relieve serious diseases?	Often	2	4
	Sometimes	2	4
	Rarely	5	10
	Never	41	82
Do you use herbal medicines to improve your quality of health?	Always	4	8
	Often	4	8
	Sometimes	16	32
	Rarely	8	16
	Never	18	36
Do you recommend consumers to use herbal medicines for self-treatment?	Always	1	2
	Often	7	14
	Sometimes	16	32
	Rarely	9	18
	Never	17	34
Do you seek scientific references with regard to herbal medicines before use or before making recommendation?	Always	10	20
	Often	6	12
	Sometimes	10	20
	Rarely	9	18
	Never	15	30

Fisher’s exact test revealed that pharmacy ownership (*p* = 0.033) as significant determinant of herbal medicine usage for self-treatment. Moreover, logistic regression showed that percentage attitude score was significantly associated (COR = 0.72, 95%CI: 0.52, 0.98, *p* = 0.036) with herbal medicine for self-treatment (Additional file 6). Variables that were significant at bivariate level were retained for multivariable level. The multivariable level revealed that both pharmacy ownership (*p* = 0.015) and percentage attitude score (*p* = 0.033) were significantly associated with usage of herbal medicine for self-treatment (Table 7). Consequently, owners were found to use herbal medicine 14.44 (AOR = 14.44, 95%CI: 1.67, 124.52) times more as compared to employee, while for a unit increase in percentage attitude score, the odds of herbal medicine usage decreased by 36.8% (AOR = 0.632, 95%CI: 0.41, 0.96).

**Table 7:** Determinants of herbal medicine usage by pharmacy professionals’ across the category of pharmacy ownership, overall knowledge and attitude scores at bivariate and multivariate levels in Asmara, Eritrea, 2021

Variable	Category	Bivariate analysis		Multivariate analysis	
		COR (95%CI)	***p***-value	AOR (95%CI)	***p***-value
**Pharmacy ownership**	Owner	6.80 (1.28, 36.27)	**0.025**	14.44 (1.67, 124.52)	**0.015**
	Employee	*Ref.*		*Ref.*	
**Overall knowledge score**	–	1.02 (0.95, 1.1)	0.533	–	–
**Attitude score**	–	0.72 (0.52, 0.98)	**0.036**	0.632 (0.41, 0.96)	**0.033**

## Discussion

In this cross-sectional study, majority of the respondents reported having used HMs for self-treatment in their lifetime even though this claim does not reveal their current use. Furthermore, in a similar study conducted in Gondar, northwest of Ethiopia, the community pharmacists did not perceive that they are authorized to dispense and that there were no regulatory provisions to oversee the sale of HMs [6]. Likewise, in this current study, a higher percentage of professionals reported that they do not recommend customers to use herbals for self-treatment which highlights the fact that those professionals are not authorized to dispense herbals and that there are no legal frameworks to do so, despite the issued policy on traditional medicine regarding the integration of complementary and alternative medicine. This study further revealed that one in five of the respondents reported seeking scientific information regarding HMs and this can be accounted to the lack of availability of accessible information on herbals; additionally, they may not feel obligated to read on the subject matter because there is no practice regarding the dispensing of HMs. A significant association was found between pharmacy ownership and prevalence of use where the owners were found to be using herbal medicines for self-treatment more than the employees. Yet, this association should be taken with care as the number of employee is four times more than the number of owners, thus making it easier for the owners to be represented in a higher proportion.

Although the association between religion and knowledge of indication of herbal medicines was found to be significant, this result needs to be considered cautiously as the number of Muslims is not comparable to that of Christian participants. The pharmacy professionals’ knowledge on indication, interaction, contra-indication and adverse events of herbal medicines was assessed to be low. This result was comparable to a similar study conducted in Ethiopia where more than half of the community pharmacists self-rated their knowledge as poor [6] and to a study conducted in Nigeria where most of the community pharmacists agreed that they did not possess adequate knowledge on the safety profiles of herbal medicines [8]. Moreover, the finding of the study was also similar with a study conducted in Lebanon where fewer pharmacists had adequate knowledge about their side effects and their interactions with drugs [9]. Overall, the reported low knowledge score, which was assessed on the basis of locally selected herbs, could be a reflection of the inadequate content of HMs information in the undergraduate courses. However, in contrast to the aforementioned studies, the researchers did not find any association between the knowledge score and the socio-demographic characteristics.

Most of the pharmacy professionals’ agreed that herbal medicines have beneficial effects. The same results have been reported in the studies conducted in Palestine (70.9%) [10] and Ethiopia (93.7%) [6]. Further, this study summarizes that the pharmacy professionals did not fail to admit to the issue of the safety concerns of herbal medicines. Additional findings regarding the pharmacy professionals’ attitude on side-effects are in line with the Ethiopian study in that more than half of the respondents of both researches disagreed that herbal medicines have fewer side effects [6]. However, in a study conducted in Palestine, 65.5% of the pharmacists’ agreed with the above statement and 82% of them regarded herbal medicines as moderately/highly safe [10]. This study also conforms to the Ethiopian study since most respondents discouraged the sale of herbal medicines in settings other than drug retail outlets [6].

This study also demonstrated that pharmacy professionals are open to the idea of educational courses on HMs centered on patient care, including dispensing and counseling as they also agreed with education on HM use and their side-effects. This would guarantee the rational use of HMs by the consumers and minimize unwanted adverse events and the risks of side effects as they also agreed with education on HM use and their side-effects. Moreover, they also recognized the need for the integration of the indigenous knowledge of herbalists and believed that workshops and trainings should be conducted in order to establish inter-professional relationship with those herbalists. They also believed that awareness raising programs targeting HMs could contribute in creating such relationships. Parallel to this outcome in a study conducted in Jordan, 80 % of respondents believed that community pharmacists need to be experts on herbal medicines and should be able to effectively counsel patients on their safe use [2].

Finally, the research did discover an inverse relationship between prevalence of use and attitude. This is to say the more prevalent the usage of herbs the lower the attitude score. This outcome can be justified with the fact that the question asked to the pharmacy professionals, in order to assess their prevalence of use, considers only if they had ever used HMs, but not about their current use, hence leading to this finding.

## Limitations

As the research was based on a cross-sectional type of study design, it did not confirm the cause-effect relationships between the determinants. Another limitation was related to the method of selection of the herbals in the study as they were all locally available plants which are commonly used among the population in Maekel (Central) region, where Asmara (the capital city of Eritrea) is located. Besides, this study is based in Asmara only and so it might not be generalizable to the entire country. Further studies at national level are required in order to get the complete picture.

## Conclusion and recommendations

This study showed that the pharmacy professionals had low knowledge score but their attitude towards HM was generally positive. In addition, herbal medicines use among pharmacy professionals has been reported to be prevalent. Therefore, the need for educational courses, trainings and workshops centered on herbal medicines has been strongly felt by the study participants.

The researchers recommend the concerned bodies to promote TM-related researches, and in particular HM-related ones and to look forward to the integration of TM into the conventional health care system while ensuring the safety of the practices. Besides, the knowledge of THPs on herbal medicines should be exploited and used for further scientific investigations. Finally, awareness enhancing programs on herbal medicines and their safety issues should be programmed by the Ministry of Health.

## Supplementary Information

**Additional file 1.** Questionnaire used for assessing the knowledge, attitude and prevalence of use of herbal medicines among pharmacy professionals working in drug retail outlet, Asmara, Eritrea.

**Additional file 2.** Determinants of knowledge on indication of herbal medicine across the categories of socio-demographic and other background characteristics at bivariate level, Asmara, Eritrea, 2021.

**Additional file 3.** Determinants of knowledge on interactions between herbal medicine and conventional medicine/dietary supplements across the categories of socio-demographic and other background characteristics at bivariate level, Asmara, Eritrea, 2021.

**Additional file 4.** Determinants of knowledge of herbal medicine on contraindications and precautions across the categories of socio-demographic and other background characteristics at bivariate level, Asmara, Eritrea, 2021.

**Additional file 5.** Determinants of knowledge of herbal medicine on adverse events and/or side-effects across the categories of socio-demographic and other background characteristics at bivariate level, Asmara, Eritrea, 2021.

**Additional file 6.** Determinants of herbal medicine usage of pharmacy professionals’ across the categories of socio-demographic and other background characteristics at bivariate level, Asmara, Eritrea, 2021.

## Data Availability

The datasets generated and/or analyzed during the current study are not publicly available due to confidentiality but are available from the corresponding author on reasonable request.

## Abbreviations

MOH: Ministry of Health
HM: Herbal Medicine
CAM: Complementary and Alternative Medicine
TMU: Traditional Medicine Unit
NMFA: National Medicine and Food Administration
TM: Traditional Medicine
THP: Traditional health practitioner/s
T&CM: Traditional and complementary medicine
ERIPA: Eritrean Pharmaceutical Association
CSPro: Census and Survey Processing System
SPSS: Statistical Package for Social Sciences
WHO: World Health Organization
