# Supplementary material for: Herbal Medicine (HM) among pharmacy professionals working in drug retail outlets in Asmara, Eritrea: knowledge, attitude and prevalence of use
Source: BMC Complement Med Ther. 2022 Aug 12;22:218. doi: 10.1186/s12906-022-03698-8 (PMC9373400; doi:10.1186/s12906-022-03698-8)
Supplement: Supplementary file 1 — Additional file 1. Questionnaire used for assessing the knowledge, attitude and prevalence of use of herbal medicines among pharmacy professionals working in drug retail outlet, Asmara, Eritrea. [file 12906_2022_3698_MOESM1_ESM.docx]

**Questionnaire on assessment of knowledge, attitude and prevalence of use of herbal medicines among pharmacy professionals working in drug retail outlets, Asmara, Eritrea: a cross-sectional study**

**Part I: Area Identification**

| Code of Drug retail outlet  Type of Drug retail outlet (Governmental/Private)  Type of Drug retail outlet (Drug shop or pharmacy)  Participant’s Serial number | \|  \|  \| \| --- \| --- \| \|  \|  \|  \|  \| \|  \|  \| \|  \|  \|  \|  \| |
| --- | --- | --- | --- | --- | --- | --- | --- | --- | --- | --- | --- | --- | --- |

| Date of interview _________/________/2021 |
| --- |
| Result of the interview 1 = Complete 2= Incomplete 3= Refused |

| Interviewer’s name and code  ___________________ | Supervisor’s name and code  ____________________ | Keyed by |
| --- | --- | --- |

**Confidentiality note: We inform the participant that any information filled in this questionnaire is confidential and will not be disclosed to anyone other than the researchers.**

**PART II: Socio-demographic and background characteristics**

| S. No | Questions | Coding Categories | Skip |
| --- | --- | --- | --- |
| 211 | Age (in completed years) |  |  |
| 212 | Sex | Male 1  Female 2 |  |
| 213 | Educational level | Diploma…………………………….1  BPharm..2  MSc3  PhD 4 |  |
| 214 | Marital status | Single 1  Married 2  Widowed 3  Separated 4 |  |
| 215 | Religion | Christian 1  Muslim 2  Others (specify) 3 |  |
| 216 | Pharmacy ownership | Owner 1  Employee 2 |  |
| 217 | Work experience in drug retail outlets (Years) |  |  |
| 218 | Overall work experience in the pharmacy field (Years) |  |  |
| 219 | Have you taken any training or workshops on herbal medicines before? | Yes 1  No 2 |  |

**PART III: Knowledge of pharmacy professionals on herbal medicine**

**Indication**

***(Multiple answers are possible)***

| S. No | Questions | Coding Categories | Skip |
| --- | --- | --- | --- |
| 311 | Aloe camperi (ሳንዳ ዕረ) | Fungal infections1  Impotency 2  Abdominal pain 3  If other, please specify…………….4  I don’t know 5 |  |
| 312 | Zingiber officinale (ጅንጂብል) | Inflammation1  Emesis2  Flatulence...3  Migraine 4  If other, please specify…………….5  I don’t know 6 |  |
| 313 | Meriandra dianthera (ንሕባ) | Hypertension 1  Diabetes2  If other, please specify…………….3  I don’t know 4 |  |
| 314 | Schinus molle (በርበረ ጸሊም) | Diarrhea 1  Common cold 2  Flu/Cough 3  Abdominal pain 4  If other, please specify…………….5  I don’t know 6 |  |
| 315 | Ruta chalepensis (ጨና ኣዳም) | Common cold 1  Flu/Cough 2  Abdominal pain 3  If other, please specify…………….4  I don’t know 5 |  |
| 316 | Senna singueana (ሃምቦሃምቦ) | Vomiting1  Loss of appetite2  Hepatitis3  If other, please specify…………….4  I don’t know 5 |  |
| 317 | Azadirachta indica (ኒም) | Hemorrhoids1  Fungal infection2  Insects and pests3  If other, please specify…………….4  I don’t know 5 |  |
| 318 | Allium sativum (ሽጉርቲ ጻዕዳ) | Hypertension1  Malaria2  Asthma3  Thrombosis4  Common cold……………………..5  Flu…………………………………6  If other, please specify…………….7  I don’t know 8 |  |
| 319 | Carica papaya (ፓፓዮ) | Diabetes 1  Amoeba infection2  Thyroid fever3  Malaria4  Constipation5  If other, please specify…………….6  I don’t know 7 |  |
| 320 | Citrus lemon (ለሚን) | Gastritis1  Digestion problems2  If other, please specify…………….4  I don’t know 3 |  |
| 321 | Aloe elegance (ዕረ) | Diabetic 1  Bacterial infection 2  If other, please specify…………….4  I don’t know 3 |  |
| 322 | Chaenopodium album (ሞቕሞቆ) | Hypertension1  Burns2  Wounds3  If other, please specify…………….4  I don’t know 5 |  |
| 323 | Trigonella foenum-graecum (ኣባዕኸ) | Hypertension 1  Diabetes2  Asthma3  Abdominal pain 4  If other, please specify…………….5  I don’t know 6 |  |

**Interactions**

| S. No | Questions | Coding Categories | Skip |
| --- | --- | --- | --- |
| 324 | Aloe camperi juice (ሳንዳ-ዕረ) reduces blood-glucose levels in patients with diabetes taking hypoglycemics such as glibenclamide. | True 1  False 2  I don’t know 3 |  |
| 325 | In the case of Allium sativum (ሽጉርቲ ጻዕዳ), a patient taking ACE inhibitors such as enalapril may develop marked hypotension and become faint after taking garlic in large amount. | True 1  False 2  I don’t know 3 |  |
| 326 | Allium sativum (ሽጉርቲ ጻዕዳ) and fish oils together may have beneficial effects on lowering blood lipids. | True 1  False 2  I don’t know 3 |  |
| 327 | Concomitant use of Senna singueana (ሃምቦሃምቦ) and conventional corticosteroids might increase the risk of hypokalemia. | True 1  False 2  I don’t know 3 |  |
| 328 | Schinus molle (በርበረ ጸሊም) might interact with theophylline. | True 1  False 2  I don’t know 3 |  |
| 329 | Zingiber officinale (ጂንጅብል) interacts with pharmacologic effects of warfarin. | True 1  False 2  I don’t know 3 |  |
| 330 | Zingiber officinale (ጂንጅብል) can decrease the metabolism of caffeine. | True 1  False 2  I don’t know 3 |  |

**Contra-indications and precautions**

| S. No | Questions | | Coding Categories | Skip |
| --- | --- | --- | --- | --- |
| 331 | Allium sativum (ሽጉርቲ ጻዕዳ) is contra-indicted in people with allergies. | | True 1  False 2  I don’t know 3 |  |
| 332 | Aloe camperi (ሳንዳ-ዕረ) should be used with precaution in patients with diabetes mellitus. | | True 1  False 2  I don’t know 3 |  |
| 333 | Zingiber officinale (ጂንጅብል) is contraindicated in individuals with gallstones. | | True 1  False 2  I don’t know 3 |  |
| 334 | Azadirachta indica (ኒም) seeds oil should not be administered to children. | | True 1  False 2  I don’t know 3 |  |
| 335 | Trigonella foenum-graecum (ኣባዓኸ) is contra-indicated in pregnancy. | True 1  False 2  I don’t know 3 | |  |

**Adverse events and/or side-effects**

**(*Multiple answers are possible*)**

| S. No | Questions | Coding Categories | Skip |
| --- | --- | --- | --- |
| 336 | Allium sativum (ሽጉርቲ ጻዕዳ) may cause | Indigestion 1  Hypersensitivity reactions (contact dermatitis and asthma) 2  I don’t know 3 |  |
| 337 | Aloe camperi juice (ሳንዳ-ዕረ) can cause; | Abdominal cramps1  Diarrhea2  I don’t know 3 |  |
| 338 | Zingiber officinale (ጂንጅብል) causes | Heartburn 1  Allergic reactions (rashes)2  I don’t know 3 |  |
| 339 | Trigonella foenum-graecum (ኣባዓኸ) causes | Abdominal distension1  Diarrhea2  Dyspepsia3  Flatulence…………………………..4  I don’t know 5 |  |
| 340 | Long term use of Azadirachta indica (ኒም) may cause; | Kidney injury 1  Liver injury 2  Miscarriage 3  Infertility……………………………4  Hypoglycemia……………………...5  I don’t know 6 |  |

**PART IV: Attitude towards herbal medicine use among pharmacy professionals**

| S. No | Questions | Coding Categories | | | | | Skip |  |
| --- | --- | --- | --- | --- | --- | --- | --- | --- |
|  |  | Strongly agree | Agree | Neutral | Disagree | Strongly disagree |  |  |
| 411 | Herbal medicines have beneficial effects. |  |  |  |  |  |  |  |
| 412 | Herbal medicines are as efficacious as conventional medicines. |  |  |  |  |  |  |  |
| 413 | Herbal medicines have placebo effects. |  |  |  |  |  |  |  |
| 414 | Herbal medicines have high qualities (in terms of active ingredients). |  |  |  |  |  |  |  |
| 421 | Herbal medicines have fewer side effects than conventional medicines. |  |  |  |  |  |  |  |
| 422 | Herbal medicines are relatively safer, because they are natural. |  |  |  |  |  |  |  |
| 423 | Herbal medicines are readily contaminated and cannot be used safely. |  |  |  |  |  |  |  |
| 424 | Herbal medicines have relatively fewer interactions in comparison with conventional medicines. |  |  |  |  |  |  |  |
| 425 | Herbal medicines have relatively fewer contra-indications in comparison with conventional medicines. |  |  |  |  |  |  |  |
| 426 | Herbal medicines have significant interactions with conventional medicine. |  |  |  |  |  |  |  |
| 431 | Pharmacy professionals are in unique position to provide evidence-based information regarding herbal medicines to help patients and customers make safe decisions about their use. |  |  |  |  |  |  |  |
| 432 | Pharmacy professionals are the right persons to advise and educate people on herbal products use. |  |  |  |  |  |  |  |
| 441 | Herbal medications (finished herbal products) should be sold only in a pharmacy. |  |  |  |  |  |  |  |
| 442 | Carrying herbal medicines may have a negative influence on a pharmacy's image. |  |  |  |  |  |  |  |
| 443 | Herbal medicines should be sold in pharmacies under a pharmacy professionals’ supervision. |  |  |  |  |  |  |  |
| 444 | Only registered pre-packaged herbal medicines should be available in community pharmacies. |  |  |  |  |  |  |  |
| 451 | Herbal medicines are a threat to public health. |  |  |  |  |  |  |  |
| 452 | Herbal medicines have a high acceptance by the public. |  |  |  |  |  |  |  |
| 453 | Herbal medicines have a positive impact on public health. |  |  |  |  |  |  |  |
| 454 | Herbal medicines should be incorporated along with the conventional medicine in the health care-service, in order to avoid self-medications by the public. |  |  |  |  |  |  |  |
| 461 | Educational courses on herbal medicines centered on patient care (counseling and dispensing) have to be provided to pharmacy professionals. |  |  |  |  |  |  |  |
| 462 | Indigenous knowledge from herbalists should be integrated for guidelines of use. |  |  |  |  |  |  |  |
| 463 | Pharmacy professionals need to be educated on herbal medicine use and their side effects. |  |  |  |  |  |  |  |
| 464 | The use of herbal medicines is an economic alternative to conventional medicines. |  |  |  |  |  |  |  |
| 465 | Workshops and trainings should be provided in order to establish inter-professional relationships between herbalists, including TMPs and pharmacy professionals. |  |  |  |  |  |  |  |

**PART V: Prevalence of use herbal medicine among pharmacy professionals**

| S. No | Questions | Coding Categories | Skip |
| --- | --- | --- | --- |
| 511 | Have you ever used herbal medicines for self-treatment? | Yes1  No…………………………………..2  I don’t remember3 |  |
| 512 | Have you ever used herbal medicines for minor ailments (common cold, scratches, tonsillitis, etc…)? | Yes1  No…………………………………..2  I don’t remember3 |  |
| 513 | Have you ever used herbal medicines to relieve some diseases on the recommendation of other conventional health professionals, a herbalist (even a TMP) or layman? | Yes1  No…………………………………..2  I don’t remember3 |  |
| 514 | Do you have any chronic disease? | Yes1  No…………………………………..2 | 516 |
| 515 | Do you use herbal medicines to manage your chronic diseases? | Always1  Often………………………………..2  Sometimes………………………….3  Rarely………………………………4  Never………………….……………5 |  |
| 516 | Do you use herbal medicines to relieve serious diseases? | Always1  Often………………………………..2  Sometimes………………………….3  Rarely………………………………4  Never………………………………5 |  |
| 517 | Do you use herbal medicines to improve your quality of health? | Always1  Often………………………………..2  Sometimes………………………….3  Rarely………………………………4  Never………………………………5 |  |
| 518 | Do you recommend consumers to use herbal medicines for self-treatment? | Always1  Often………………………………..2  Sometimes………………………….3  Rarely………………………………4  Never……………………………….5 |  |
| 519 | Do you seek scientific references with regard to herbal medicines before use or before making recommendation? | Always1  Often………………………………..2  Sometimes………………………….3  Rarely………………………………4  Never……………………………….5 |  |

**Comment section**

____________________________________________________________________________________________________________________________________________________________________________________________________________________________________________________________________________________________________________________________________________________________________________________________________________________________________________________________________________________
