# Supplementary material for: Herbal Medicine (HM) among pharmacy professionals working in drug retail outlets in Asmara, Eritrea: knowledge, attitude and prevalence of use
Source: BMC Complement Med Ther. 2022 Aug 12;22:218. doi: 10.1186/s12906-022-03698-8 (PMC9373400; doi:10.1186/s12906-022-03698-8)
Supplement: Supplementary file 2 — Additional file 2. Determinants of knowledge on indication of herbal medicine across the categories of socio-demographic and other background characteristics at bivariate level, Asmara, Eritrea, 2021. [file 12906_2022_3698_MOESM2_ESM.docx]

**Determinants of knowledge on indication of herbal medicine across the categories of socio-demographic and other background characteristics at bivariate level, Asmara, Eritrea, 2021**

| **Variable** | **Coding category** | **Median (IQR)** | **Mann-Whitney Z/**  **Kruskal-Wallis χ^2^** | ***p*-value** |
| --- | --- | --- | --- | --- |
| Type of pharmacy retail outlets by privacy | Governmental | 30.85 (18.09) | -1.75 | 0.080 |
|  | Private | 25.53 (10.64) |  |  |
| Type of pharmacy retail outlets | Drug shop | 23.40 (9.04) | -0.54 | 0.568 |
|  | Pharmacy | 27.66 (15.43) |  |  |
| Sex | Male | 25.53 (10.64) | -1.06 | 0.288 |
|  | Female | 29.79 (17.02) |  |  |
| Religion | Christian | 27.66 (15.43) | -2.44 | **0.015** |
|  | Muslim | 15.96 (8.51) |  |  |
| Educational level | Diploma | 27.66 (10.64) | 0.19 | 0.909 |
|  | Degree | 27.66 (16.49) |  |  |
|  | Masters | 25.53 (-)* |  |  |
| Marital status | Single | 29.79 (18.09) | 0.27 | 0.873 |
|  | Married | 26.60 (10.64) |  |  |
|  | Separated | 25.53 (-)* |  |  |
| Pharmacy ownership | Owner | 25.53 (12.77) | -1.29 | 0.196 |
|  | Employee | 27.66 (17.02) |  |  |
| Training or workshop on herbal medicines | Yes | 24.47 (10.11) | -0.64 | 0.519 |
|  | No | 27.66 (14.89) |  |  |
| **Variables** | | | **r_s_** | ***p*-value** |
| Age | | | -0.154 | 0.287 |
| Work experience (in retail pharmacy outlet) | | | -0.154 | 0.284 |
| Overall work experience (pharmacy field) | | | -0.262 | 0.066 |
