# Supplementary material for: Herbal Medicine (HM) among pharmacy professionals working in drug retail outlets in Asmara, Eritrea: knowledge, attitude and prevalence of use
Source: BMC Complement Med Ther. 2022 Aug 12;22:218. doi: 10.1186/s12906-022-03698-8 (PMC9373400; doi:10.1186/s12906-022-03698-8)
Supplement: Supplementary file 3 — Additional file 3. Determinants of knowledge on interactions between herbal medicine and conventional medicine/dietary supplements across the categories of socio-demographic and other background characteristics at bivariate level, Asmara, Eritrea, 2021. [file 12906_2022_3698_MOESM3_ESM.docx]

**Determinants of knowledge on interactions between herbal medicine and conventional medicine/dietary supplements across the categories of socio-demographic and other background characteristics at bivariate level, Asmara, Eritrea, 2021**

| **Variable** | **Coding category** | **Median (IQR)** | **Mann-Whitney Z/**  **Kruskal-Wallis χ^2^** | ***p*-value** |
| --- | --- | --- | --- | --- |
| Type of drug retail outlets by privacy | Governmental | 28.57 (28.57) | -1.28 | 0.20 |
|  | Private | 14.29 (32.14) |  |  |
| Type of drug retail outlets | Drug shop | 14.29 (28.57) | -0.99 | 0.321 |
|  | Pharmacy | 28.57 (42.86) |  |  |
| Sex | Male | 28.57 (42.86) | -0.31 | 0.758 |
|  | Female | 14.29 (28.57) |  |  |
| Religion | Christian | 14.29 (42.86) | -0.37 | 0.70 |
|  | Muslim | 28.57 (32.14) |  |  |
| Educational level | Diploma | 28.57 (28.57) | 0.15 | 0.929 |
|  | BPharm | 21.43 (42.86) |  |  |
|  | MSc | 14.29 (-)* |  |  |
| Marital status | Single | 28.57 (28.57) | 1.48 | 0.477 |
|  | Married | 28.57 (42.86) |  |  |
|  | Separated | 14.29 (-)* |  |  |
| Pharmacy ownership | Owner | 28.57 (28.57) | -0.97 | 0.331 |
|  | Employee | 14.29 (35.71) |  |  |
| Training or workshop on herbal medicines | Yes | 28.57 (42.86) | -0.25 | 0.806 |
|  | No | 21.43 (28.57) |  |  |
| **Variables** | | | **r_s_** | ***p*-value** |
| Age | | | -0.195 | 0.174 |
| Work experience (in drug retail outlet) | | | -0.140 | 0.333 |
| Overall work experience (pharmacy field) | | | -0.213 | 0.138 |
| *Note: IQR: Interquartile range, Z: Z score;*: Too few data to get the IQR,* χ^2^: Chi-square, r_s_: Spearman rank correlation | | | | |
