# Supplementary material for: Herbal Medicine (HM) among pharmacy professionals working in drug retail outlets in Asmara, Eritrea: knowledge, attitude and prevalence of use
Source: BMC Complement Med Ther. 2022 Aug 12;22:218. doi: 10.1186/s12906-022-03698-8 (PMC9373400; doi:10.1186/s12906-022-03698-8)
Supplement: Supplementary file 4 — Additional file 4. Determinants of knowledge of herbal medicine on contraindications and precautions across the categories of socio-demographic and other background characteristics at bivariate level, Asmara, Eritrea, 2021. [file 12906_2022_3698_MOESM4_ESM.docx]

**Determinants of knowledge of herbal medicine on contraindications and precautions across the categories of socio-demographic and other background characteristics at bivariate level, Asmara, Eritrea, 2021**

| **Variable** | **Coding category** | **Median (IQR)** | **Mann-Whitney Z/**  **Kruskal-Wallis χ^2^** | ***p*-value** |
| --- | --- | --- | --- | --- |
| Type of drug retail outlets by privacy | Governmental | 40 (35) | -0.26 | 0.794 |
|  | Private | 40 (40) |  |  |
| Type of drug retail outlets | Drug shop | 40 (30) | -0.72 | 0.472 |
|  | Pharmacy | 40 (40) |  |  |
| Sex | Male | 40 (40) | -0.08 | 0.933 |
|  | Female | 40 (20) |  |  |
| Religion | Christian | 40 (40) | -0.64 | 0.526 |
|  | Muslim | 30 (20) |  |  |
| Educational level | Diploma | 40 (40) | 2.27 | 0.322 |
|  | Degree | 40 (20) |  |  |
|  | Masters | 20 (-)* |  |  |
| Marital status | Single | 20 (40) | 0.93 | 0.628 |
|  | Married | 40 (25) |  |  |
|  | Separated | 20 (-)* |  |  |
| Pharmacy ownership | Owner | 40 (30) | -0.08 | 0.937 |
|  | Employee | 40 (40) |  |  |
| Training or workshop on herbal medicines | Yes | 40 (45) | -0.76 | 0.445 |
|  | No | 40 (35) |  |  |
| **Variables** | | | **r_s_** | ***p*-value** |
| Age | | | 0.086 | 0.553 |
| Work experience (in drug retail outlet) | | | -0.018 | 0.901 |
| Overall work experience (pharmacy field) | | | -0.013 | 0.926 |
| *Note: IQR: Interquartile range, Z: Z score;*: Too few data to get the IQR,* χ^2^: Chi-square, r_s_: Spearman rank correlation | | | | |
