# Supplementary material for: Herbal Medicine (HM) among pharmacy professionals working in drug retail outlets in Asmara, Eritrea: knowledge, attitude and prevalence of use
Source: BMC Complement Med Ther. 2022 Aug 12;22:218. doi: 10.1186/s12906-022-03698-8 (PMC9373400; doi:10.1186/s12906-022-03698-8)
Supplement: Supplementary file 5 — Additional file 5. Determinants of knowledge of herbal medicine on adverse events and/or side-effects across the categories of socio-demographic and other background characteristics at bivariate level, Asmara, Eritrea, 2021. [file 12906_2022_3698_MOESM5_ESM.docx]

**Determinants of knowledge of herbal medicine on adverse events and/or side-effects across the categories of socio-demographic and other background characteristics at bivariate level, Asmara, Eritrea, 2021**

| **Variable** | **Coding category** | **Median (IQR)** | **Mann-Whitney Z/**  **Kruskal-Wallis χ^2^** | ***p*-value** |
| --- | --- | --- | --- | --- |
| Type of drug retail outlets | Governmental | 16.67 (20) | -0.98 | 0.325 |
|  | Private | 13.33 (13.33) |  |  |
| Type of drug retail outlets | Drug shop | 13.33 (11.67) | -0.09 | 0.925 |
|  | Pharmacy | 13.33 (20) |  |  |
| Sex | Male | 13.33 (20) | -0.66 | 0.509 |
|  | Female | 13.33 (13.33) |  |  |
| Religion | Christian | 13.33 (20) | -0.11 | 0.913 |
|  | Muslim | 16.67 (11.67) |  |  |
| Educational level | Diploma | 13.33 (6.67) | 1.1 | 0.586 |
|  | BPharm | 13.33 (20) |  |  |
|  | MSc | 6.67 (-)* |  |  |
| Marital status | Single | 29.79 (18.09) | 1.54 | 0.463 |
|  | Married | 26.60 (10.64) |  |  |
|  | Separated | 25.53 (-)* |  |  |
| Pharmacy ownership | Owner | 13.33 (20) | -0.87 | 0.383 |
|  | Employee | 13.33 (20) |  |  |
| Training or workshop on herbal medicines | Yes | 13.33 (16.67) | -0.12 | 0.903 |
|  | No | 13.33 (20) |  |  |
| **Variables** | | | **r_s_** | ***p*-value** |
| Age | | | -0.019 | 0.898 |
| Work experience (in drug retail outlet) | | | -0.057 | 0.692 |
| Overall work experience (pharmacy field) | | | -0.082 | 0.570 |
| *Note: IQR: Interquartile range, Z: Z score;*: Too few data to get the IQR,* χ^2^: Chi-square, r_s_: Spearman rank correlation | | | | |
