# Supplementary material for: Herbal Medicine (HM) among pharmacy professionals working in drug retail outlets in Asmara, Eritrea: knowledge, attitude and prevalence of use
Source: BMC Complement Med Ther. 2022 Aug 12;22:218. doi: 10.1186/s12906-022-03698-8 (PMC9373400; doi:10.1186/s12906-022-03698-8)
Supplement: Supplementary file 6 — Additional file 6. Determinants of herbal medicine usage of pharmacy professionals’ across the categories of socio-demographic and other background characteristics at bivariate level, Asmara, Eritrea, 2021. [file 12906_2022_3698_MOESM6_ESM.docx]

**Determinants of herbal medicine usage of pharmacy professionals’ across the categories of socio-demographic and other background characteristics at bivariate level, Asmara, Eritrea, 2021**

| **Variable** | **Coding category** | **Ever used herbal medicine for self-treatment** | | ***p***-**value*** |
| --- | --- | --- | --- | --- |
|  |  | **Yes n (%)** | **No n (%)** |  |
| Type of drug retail outlets by privacy | Governmental | 13 (86.7) | 2 (13.3) | 1.000 |
|  | Private | 26 (81.3) | 6 (18.8) |  |
| Type of drug retail outlets | Drug shop | 5 (83.3) | 1 (16.7) | 1.000 |
|  | Pharmacy | 34 (82.9) | 7 (17.1) |  |
| Sex | Male | 23 (79.3) | 6 (20.7) | 0.692 |
|  | Female | 16 (88.9) | 2 (11.1) |  |
| Religion | Christian | 36 (81.8) | 8 (18.2) | 1.000 |
|  | Muslim | 3 (100) | 0 (0) |  |
| Educational level | Diploma | 11 (91.7) | 1 (8.3) | 0.421 |
|  | BPharm | 26 (81.3) | 6 (18.8) |  |
|  | MSc | 2 (66.7) | 1 (33.3) |  |
| Marital status | Single | 11 (91.7) | 1 (8.3) | 0.673 |
|  | Married | 25 (78.1) | 7 (21.9) |  |
|  | Separated | 3 (100) | 0 (0) |  |
| Pharmacy ownership | Owner | 5 (55.6) | 4 (44.4) | 0.033 |
|  | Employee | 34 (89.5) | 4 (10.5) |  |
| Training or workshop on herbal medicines | Yes | 5 (83.3) | 34 (82.9) | 1.000 |
|  | No | 1 (16.7) | 7 (17.1) |  |
| ***p***-**value*: fisher’s exact test was used** | | | | |
